# Neuromelanin magnetic resonance imaging of substantia nigra and locus coeruleus in Parkinson’s disease with freezing of gait

**DOI:** 10.3389/fnagi.2023.1060935

**Published:** 2023-02-02

**Authors:** Shangpei Wang, Tong Wu, Yajie Cai, Yongqiang Yu, Xianwen Chen, Longsheng Wang

**Affiliations:** ^1^Department of Radiology, The Second Hospital of Anhui Medical University, Hefei, Anhui, China; ^2^Department of Neurology, The First Affiliated Hospital of Anhui Medical University, Hefei, Anhui, China; ^3^Department of Radiology, The First Affiliated Hospital of Anhui Medical University, Hefei, Anhui, China

**Keywords:** Parkinson’s disease, freezing of gait, neuromelanin MRI, substantia nigra, locus coeruleus

## Abstract

**Background:**

The downregulation of monoamines, especially dopamine in substantia nigra (SN) and norepinephrine in locus coeruleus (LC), may be responsible for freezing of gait (FOG) pathological basis in Parkinson’s disease (PD).

**Methods:**

Thirty-two Parkinson’s disease patients with freezing of gait (PD-FOG), 32 Parkinson’s disease patients without freezing of gait (PD-NFOG) and 32 healthy controls (HC) underwent neuromelanin magnetic resonance imaging (NM-MRI). The volume, surface area and contrast to noise ratio (CNR) of SN and LC were measured and compared. The correlation analyses were conducted between the measurements of SN and LC with clinical symptoms. We plotted the receiver operating characteristic (ROC) curve and determined the sensitivity and specificity of the CNR of SN and LC for discriminating the PD-FOG from the PD-NFOG.

**Results:**

Both PD-FOG and PD-NFOG showed decreased volume, surface area and CNR of SN compared with HC. The PD-FOG exhibited decreased volume and surface area of LC compared with both PD-NFOG and HC groups, and decreased CNR of LC compared with HC group. The volume, surface area and CNR of SN were negatively correlated with the Unified Parkinson’s Disease Rating Scale part III scores. The illness durations in PD patients were negatively correlated with the volume, surface area of SN, while not the CNR. And the volume and surface area of LC were negatively correlated with new freezing of gait questionnaire scores. ROC analyses indicated that the area under the curve (AUC) was 0.865 and 0.713 in the CNR of SN and LC, respectively, in PD versus HC, whereas it was 0.494 and 0.637 respectively, in PD-FOG versus PD-NFOG. Among these, for discriminating the PD from the HC, the sensitivity and specificity in the CNR of the SN was 90.6 and 71.9%, respectively, when the cut-off value was set at 2.101; the sensitivity and specificity in the CNR of the LC was 90.6 and 50.0%, respectively, when the cut-off value for CNR was set at 1.411.

**Conclusion:**

The dopaminergic changes in the SN were found across both PD-FOG and PD-NFOG, whilst LC noradrenergic neuron reduction was more evident in PD-FOG.

## 1. Introduction

Parkinson’s disease (PD) is one of the most common neurodegenerative diseases and characterized by the loss of dopaminergic neurons in the substantia nigra (SN) of the midbrain and the presence of Lewy bodies (Kalia and Lang, 2015). SN degeneration causes a decrease of dopamine in the striatum, resulting in dysfunction of the motor control system including the basal ganglia, leading to rest tremor, muscular rigidity, bradykinesia and gait impairment. There is also massive loss of neurons in the locus coeruleus (LC) with 83% of an average loss rate in the late stage of PD according to postmortem reports (Sulzer and Surmeier, 2013b). The decreased content of noradrenaline may cause freezing of gait (FOG), autonomic nervous system dysfunction, depression, sleep disorder and other symptoms (Fearnley and Lees, 1991; Jost, 2003).

FOG is one of the most common motor symptoms experienced by PD patients and is defined as a brief, sudden inability to initiate walking despite the intention to step. FOG reduces mobility and significantly increases the risk of falls, resulting in devastating consequences. Even though dysfunction of the acetylcholine, norepinephrine and dopamine is considered to relate to FOG, the neural mechanisms are poorly understood. And the downregulation of monoamines, especially dopamine (Giladi, 2008) and norepinephrine (Freed et al., 2001), may also be responsible for the pathological basis of FOG. The concentrations of noradrenaline and dopamine synthetase such as dopamine hydroxylase decreased in brain tissue of PD patients (Tohgi et al., 1993). In addition, noradrenaline concentrations in the cerebrospinal fluid (CSF) are related with FOG (Nagatsu et al., 1977). Recent animal studies have indicated dysfunction in the noradrenergic pathway, not only in the dopaminergic pathway, also leads to dyskinesia (Rommelfanger et al., 2007; Ebersbach et al., 2013; Espay et al., 2014). Clinical observations show that noradrenaline precursor could partially improve FOG symptoms as well (Fukada et al., 2013).

Noradrenergic transmissions from the LC play a major role in the regulation of brain functions, and project to the extensive motor and sensory cortex. Behavioral studies suggest that release of noradrenaline in response to a particular sensory event will provoke or facilitate dynamic reorganization of neural networks, creating completely new functional networks (Bouret and Sara, 2005). This functional reconfiguration will govern the adaptive behavioral output. Previous studies have shown that FOG is associated with the decreased local metabolism of noradrenergic neurons (Ono et al., 2016) and the loss of projection from the LC to the frontal lobe (Espay et al., 2014). Thus, LC dysfunction may be one of potential mechanisms in the FOG onset (Shine et al., 2011; Smulders et al., 2015).

Molecular imaging technology such as single-photon emission computed tomography (SPECT) provides multiple methods for PD diagnosis (Nikolaus et al., 2009; la Fougere et al., 2010). Dopamine transporter and ^123^I-metaiodobenzylguanidine SPECT are wildly used in this area. However, considering the imaging technology with an indirectly brain function evaluation can be regulated by dopamine replacement therapy, decreased uptake does not necessarily represent the extent of neurodegeneration. With the development of magnetic resonance image (MRI) technology, the alterations of the SN and LC corresponding to the pathological results are still hard to be depicted directly (Bae et al., 2021). Previous studies (Bartzokis et al., 1999; Vymazal et al., 1999; Graham et al., 2000; Martin et al., 2008) have reported an increase of iron content according to quantifying T_2_ weighted imaging (T_2_WI), but with controversial results. It may be contributed by huge individual differences, complicated reconstruction process and nonlinear relationship between iron accumulations with cell degenerations (Gałazka-Friedman and Friedman, 1997). As T_2_WI could not detected monoaminergic neurons in gross specimens satisfactorily (Oikawa et al., 2002), quantitative susceptibility mapping (QSM), the derivate of T_2_WI, reported a better diagnostic accuracy and discrimination power in basal nuclei but merely including the SN not LC (Li et al., 2020).

Similar to skin melanin, neuromelanin is an oxidative product of dopamine and norepinephrine metabolism (Zucca et al., 2006) and considered to be a major storage form of iron ions in neurons (Double et al., 2003; Zecca et al., 2008). Pathological studies have indicated a significant reduction of neuromelanin in PD patients (Martin-Bastida et al., 2017). As iron ions have paramagnetic T_1_ shortening effects, recently developed sequence technology detecting contrast is called neuromelanin-MRI (NM-MRI) (Enochs et al., 1997; Sasaki et al., 2006; Chen et al., 2014). Previous NM-MRI studies quantitatively reflecting the survival of neurons both in SN and LC also have reported PD patients showed a significant decreased signal (Reimao et al., 2015a; Schwarz et al., 2017). And the high signal strength of SN is correlated with the quantity of neuromelanin-containing neurons (Kitao et al., 2013). The aim of our study is to investigate the NM-MRI features of SN and LC in PD patients with FOG (PD-FOG) and possible differences compared with PD patients without FOG (PD-NFOG).

## 2. Materials and methods

### 2.1. Participants

Sixty-four PD patients, including thirty-two PD-FOG and thirty-two PD-NFOG were recruited from the inpatient and outpatient departments of The First Affiliated Hospital of Anhui Medical University. The diagnoses of PD participants were clinically determined by a movement disorders specialist according to Movement Disorder Society Clinical Diagnostic Criteria for PD (Postuma et al., 2015). Participants were included in PD-FOG group only if FOG episodes were naturally happened or induced by provocative test in the consulting room. Exclusion criteria included MRI contraindications, presence of focal brain lesions on MRI, atypical parkinsonian disorders, previous history of other neurological conditions, psychiatric disorders or musculoskeletal impairments that interfere with gait or balance, major medical illness within the previous three months and inability to follow instructions. The PD-FOG patients were matched for age, sex and mini mental status examination score (MMSE) with PD-NFOG patients. A cohort of thirty-two age- and sex-matched healthy controls (HC), without neuropsychiatric comorbidities or any family histories of parkinsonism were recruited. All the subjects recruited for this study were right-handed. Parkinsonian motor symptoms were assessed using the Hoehn-Yahr staging scale and the Unified Parkinson’s Disease Rating Scale part III (UPDRS III). Next, the new freezing of gait questionnaire (NFOGQ) (Nieuwboer et al., 2009) was used to quantitatively assess the severity of FOG. All tests were conducted after withholding anti-parkinsonian drugs greater than 12 h, in the practical ‘Off’ levodopa state. The anti-parkinsonian dosages are reported as levodopa equivalents, which were calculated based on clinically equivalent dosing estimates (Tomlinson et al., 2010). For each PD patient, the levodopa equivalent daily dose (LEDD) was estimated based on the anti-Parkinsonian drugs and dosages used in the week before the MRI scan. The investigation was conducted in accordance with the Declaration of Helsinki and was approved by the Ethics Committee of the First Affiliated Hospital of Anhui Medical University. The participants provided informed written consent to participate in the study.

### 2.2. Magnetic resonance imaging protocol

All MR data were acquired using a 3.0 Tesla system (Discovery 750W; GE Healthcare, Milwaukee, WI, United States) equipped with the 24-channel head and neck combined coil. NM-MRI was performed using a T_1_ weighted fast spin echo sequence. The parameters used were as follows: repetition time (TR) = 600 ms; echo time (TE) = 13 ms; flip angle (FA) = 111°; echo train length = 2; matrix size = 512 × 320; field of view (FOV) = 220 × 220 mm^2^; axial slice number = 16; slice thickness = 2.5 mm, no gap; NEX = 5; and total time of acquisition = 8 min and 3 s. The axial planes were set perpendicular to the floor of the fourth ventricle with coverage from the posterior commissure to the pons. In addition, T_2_ fluid-attenuated inversion recovery images were obtained to exclude other pathological imaging findings that might interfere with further imaging assessment. All images were visually inspected for the movement artifacts before the follow-up measurements.

### 2.3. Measurements of the neuromelanin sensitive substantia nigra and locus coeruleus

**Figure 1:**
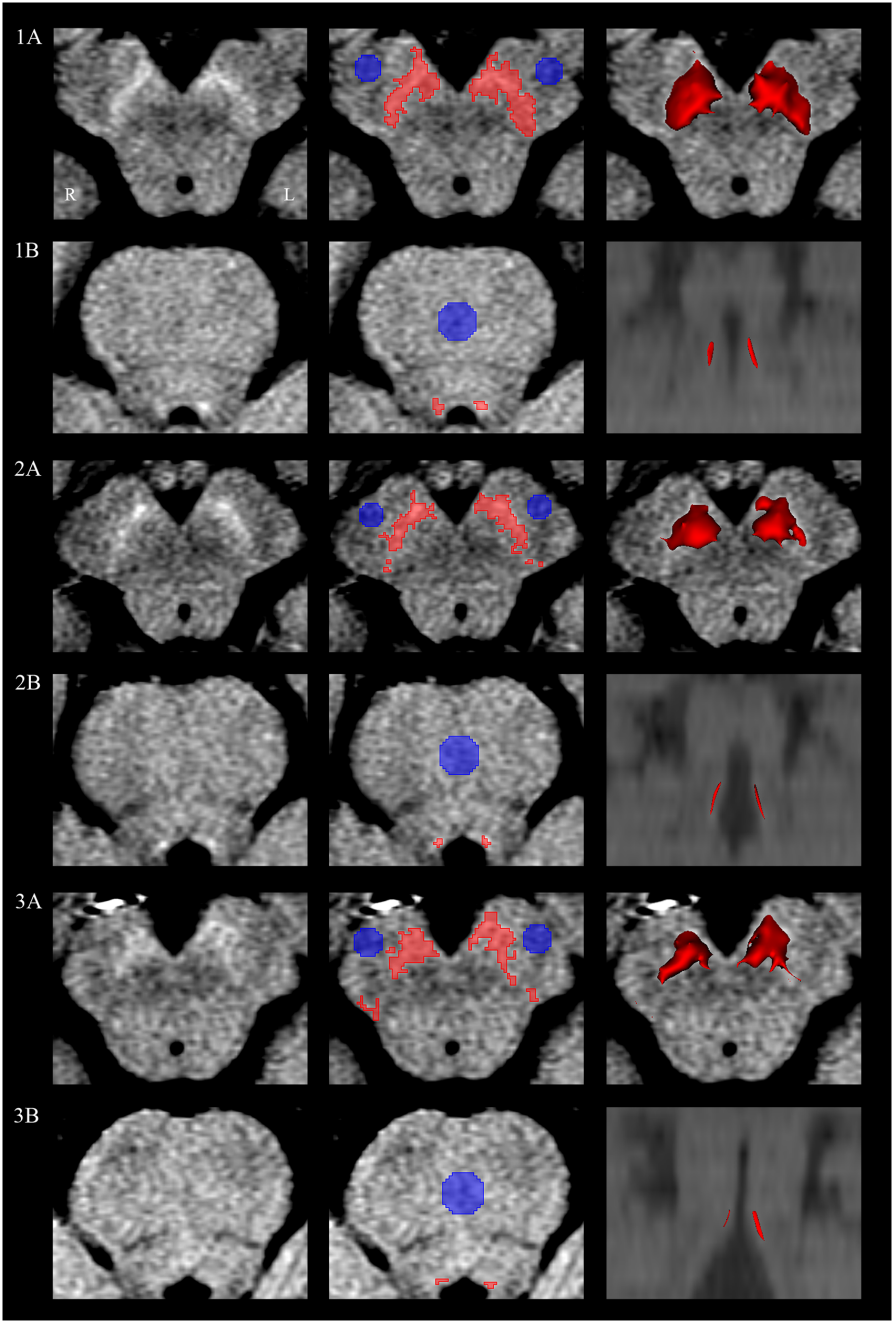
Semiautomated interactively segmented regions of interest on neuromelanin MRI. Substantia nigra and locus coeruleus of a HC subject (**1A**,**1B**), PD-NFOG patient (**2A**,**2B**) and PD-FOG patient (**3A**,**3B**). Neuromelanin MRI images of the substantia nigra and locus coeruleus (first column). Regions of interest of substantia nigra and locus coeruleus detected in the semiautomated segmentation shown in red, background reference regions of interest of cerebral peduncle and pontine shown in blue (second column). Three-dimensional surface rendering corresponding to semiautomated segmentation of the bilateral substantia nigra and locus coeruleus (third column). L, left; MRI, magnetic resonance imaging; R, right.

The 3D Slicer software package (version 4.11)^1^ was used to measure the SN and LC. The SN was visible for four consecutive slices below the cross-section of the inferior colliculus. Similar to previous studies (Langley et al., 2015; Isaias et al., 2016; Wang et al., 2018; Li et al., 2019), the level tracing segment editor module was used to create regions of interest (ROIs) semiautomated interactively in a blind manner by a neuroradiologist with 9 years of experience (SW). The volume and surface area of SN and LC were then calculated. ROIs for background were defined as circular areas (4 mm in diameter) in the cerebral peduncle (CP) on the left and right sides (Figure 1). The contrast-to-noise ratio (CNR) between the SN and CP was then calculated with the following equation: CNR_SN_ = (SI_SN_–SI_CP_)/SD_CP_. SI_SN_ and SI_CP_ represented the mean signal intensity in the ROIs for SN and CP, respectively, and SD_CP_ represented the standard deviation of the ROI for CP. The values of SI_CP_ and SD_CP_ used in the equation were the average values of both CP sides.

The location of LC was identified as the spot with the highest intensity adjacent to the fourth ventricle on the bilateral sides, which was visible for three axial slices at the level of the pons. Similar to the SN assessment, the volume and surface area of LC were assessed semiautomatically. Background reference ROIs (circles with a 6 mm in diameter) were placed in the pontine (PT) tegmentum. We calculated the CNR between the LC and PT according to the following equation: CNR_LC_ = (SI_LC_–SI_PT_)/SD_PT_. SI_LC_ and SI_PT_ represent the mean signal intensity in the ROIs for LC and PT, respectively, and SD_PT_ represents the standard deviation of the ROI for PT.

### 2.4. Statistical analysis

The demographic and clinical data were analyzed using the statistical package for the social sciences version 19.0 (SPSS, Chicago, IL, United States). Group difference in gender was tested using a chi-square test. Age and MMSE were compared between PD-FOG, PD-NFOG and HC using a one-way analysis of variance (ANOVA). Illness duration, UPDRS III and LEDD were compared between PD-FOG and PD-NFOG by using two sample *t*-test. Hoehn-Yahr stage was compared between PD-FOG and PD-NFOG using a Mann–Whitney *U* test for ranked data. Group differences in volume, surface area and CNR among the three groups were tested using a one-way ANOVA with *post hoc* intergroup comparisons. Pearson’s correlation coefficients were calculated to examine the associations between the volume, surface area and CNR and illness duration, UPDRS III and NFOGQ. In order to investigate the inter-rater agreement, NM-MRI images were measured again by another radiologist (YC) who was blind to subjects’ statues and the intra-class correlation coefficient (ICC) values of CNR, the relative parameter, were computed. Additionally, we plotted the receiver operating characteristic (ROC) curve, calculated the Youden index of the CNR and attempted to find the cut-off value to discriminate the PD and the HC, and the PD-FOG and the PD-NFOG.

## 3. Results

### 3.1. Demographic and clinical characteristics

Demographic and clinical data for the subjects were presented in Table 1. The three groups were well-matched in sex (chi-square test, *χ^2^* = 0.762, *p* = 0.683), age (one-way ANOVA, *F* = 0.217, *p* = 0.805), and MMSE (one-way ANOVA, *F* = 2.024, *p* = 0.138). There were no significant differences in illness durations (two sample *t*-test, *t* = 1.667, *p* = 0.101), Hoehn-Yahr stage (Mann–Whitney *U* test, *Z* = −1.632, *p* = 0.103), UPDRS III (two sample *t*-test, *t* = 1.603, *p* = 0.114), and LEDD (two sample *t*-test, *t* = 1.858, *p* = 0.068) between PD-FOG and PD-NFOG groups.

**Table 1:** Demographic and clinical characteristics.

Characteristics	PD-FOG (*n* = 32)	PD-NFOG (*n* = 32)	HC (*n* = 32)	Statistics	*p-*Value
Sex (male/female)	13/19	13/19	16/16	*χ^2^* = 0.762	0.683^a^
Age (years)	63.938 ± 9.698	64.344 ± 8.377	62.906 ± 8.866	*F* = 0.217	0.805^b^
MMSE	25.594 ± 2.589	26.656 ± 2.391	26.563 ± 1.999	*F* = 2.024	0.138^c^
Illness duration (years)	5.781 ± 3.590	4.484 ± 2.545	NA	*t* = 1.667	0.101^c^
Hoehn-Yahr stage	2.344 ± 0.701	2.016 ± 0.678	NA	*Z* = −1.632	0.103^d^
UPDRS III	32.156 ± 15.992	26.719 ± 10.599	NA	*t* = 1.603	0.114^c^
LEDD (mg)	392.188 ± 191.391	314.453 ± 139.162	NA	*t* = 1.858	0.068^c^
NFOGQ	20.970 ± 5.127	NA	NA	NA	NA

The data are shown as the mean values ± standard deviations. NA, not applicable; UPDRS III, Unified Parkinson’s Disease Rating Scale part III; LEDD, Levodopa equivalent daily dose; MMSE, mini-mental state examination; NFOGQ, New Freezing of Gait Questionnaire; PD-FOG, Parkinson’s disease with freezing of gait; PD-NFOG, Parkinson’s disease without freezing of gait; HC, Healthy controls.

a Chi-square test was used to test the difference in sex across three groups.

b One-way ANOVA was used to test the difference in age across three groups.

c Two-sample *t*-test was used to compare the differences in MMSE, illness duration, UPDRS III and LEDD between PD-FOG and PD-NFOG.

d Mann–Whitney *U* test was used to compare Hoehn-Yahr stage between PD-FOG and PD-NFOG.

### 3.2. Analysis of SN and LC

**Figure 2:**
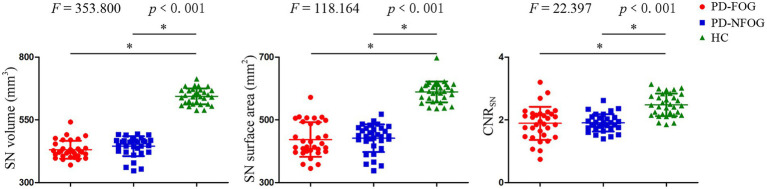
Substantia nigra volume, surface area and CNR among PD-FOG, PD-NFOG, and HC groups. CNR, contrast to noise ratio; SN, substantia nigra; *p < 0.001.

**Figure 3:**
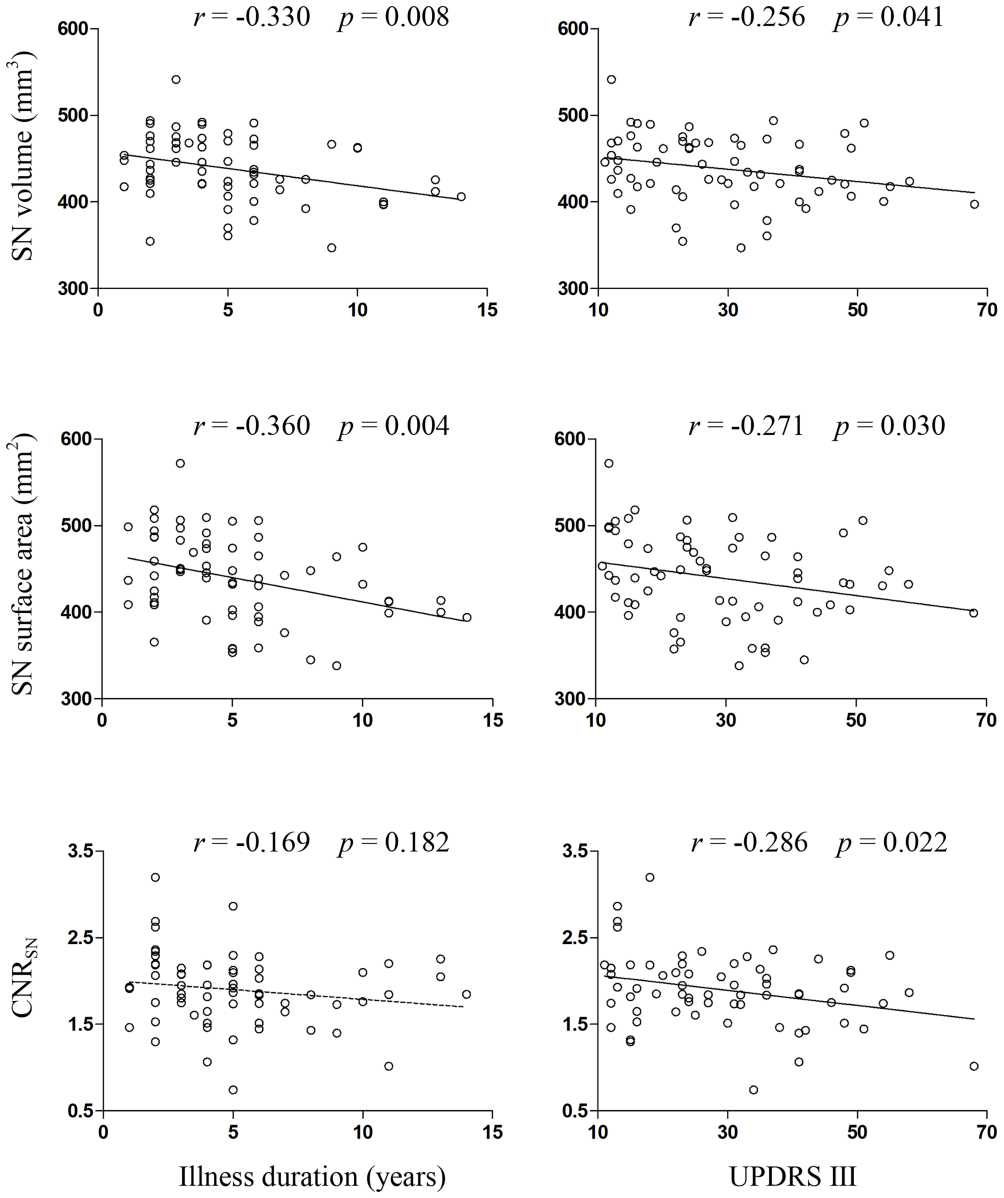
Correlations between measures of SN and clinical variables in PD patients. CNR, contrast to noise ratio; SN, substantia nigra; UPDRS III, Unified Parkinson’s Disease Rating Scale part III.

Compared with the HC group, both PD groups showed a decreased volume, surface area and CNR_SN_, which were defined as common SN alterations shared by PD-FOG and PD-NFOG (Table 2; Figure 2). Correlation analysis were performed for the volume, surface area and CNR_SN_ with illness duration and UPDRS III in all the PD patients (Figure 3). Significant correlations were observed between the volume and surface area of SN and illness duration of the PD patients, while no significant correlation was observed between the CNR_SN_ and illness duration. The volume, surface area and CNR_SN_ were significantly correlated with the UPDRS III of the PD patients.

**Table 2:** Measures of SN and LC in PD-FOG, PD-NFOG and HC groups.

	PD-FOG	PD-NFOG	HC	Statistics	*p* value
SN volume (mm^3^)	431.111 ± 35.890	445.230 ± 39.480	643.779 ± 31.471	*F* = 353.800	<0.001
SN surface area (mm^2^)	437.259 ± 54.994	441.413 ± 43.999	589.020 ± 33.329	*F* = 118.164	<0.001
CNR_SN_	1.890 ± 0.527	1.903 ± 0.277	2.478 ± 0.359	*F* = 22.397	<0.001
LC volume (mm^3^)	18.334 ± 1.050	23.138 ± 1.315	23.484 ± 1.396	*F* = 166.405	<0.001
LC surface area (mm^2^)	6.928 ± 2.427	11.487 ± 4.080	12.955 ± 4.364	*F* = 22.805	<0.001
CNR_LC_	1.470 ± 0.598	1.699 ± 0.568	1.982 ± 0.631	*F* = 5.863	0.004

The data are shown as the mean values ± standard deviations. CNR, contrast-to-noise ratio; HC, healthy controls; LC, locus coeruleus; PD-FOG, Parkinson’s disease with freezing of gait; PD-NFOG, Parkinson’s disease without freezing of gait; SN, substantia nigra.

**Figure 4:**
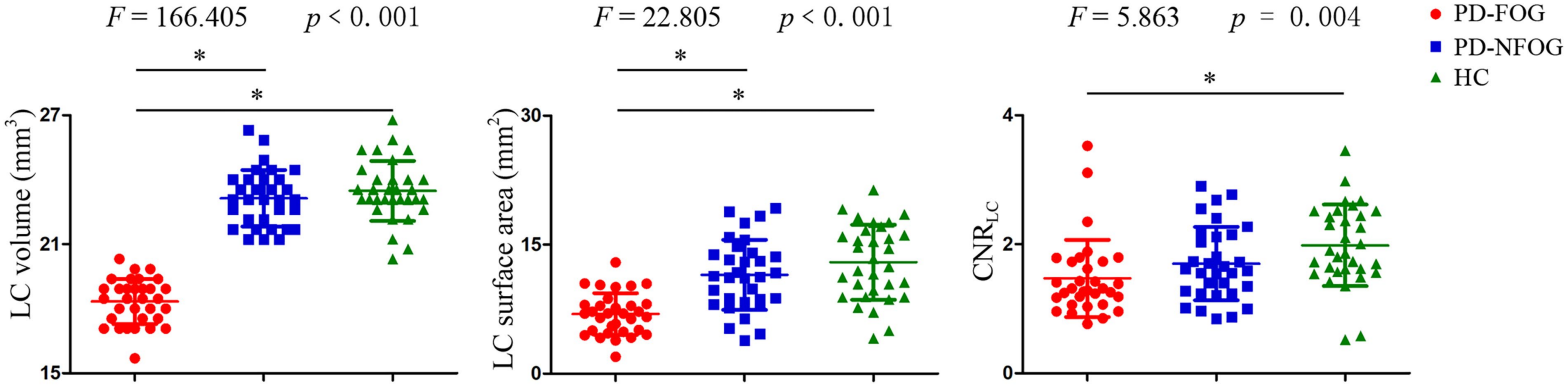
LC volume, surface area and CNR among PD-FOG, PD-NFOG and HC groups. CNR, contrast to noise ratio; LC, locus coeruleus; *p ≤ 0.001.

**Figure 5:**
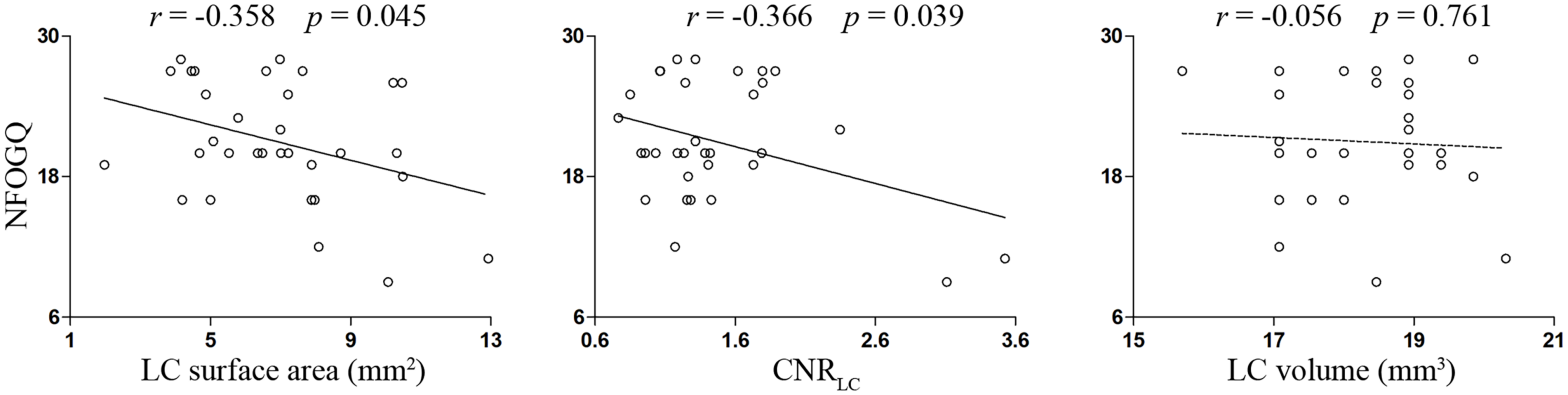
Correlations between measures of LC and NFOGQ in PD-FOG patients. CNR, contrast to noise ratio; LC, locus coeruleus; NFOGQ, new freezing of gait questionnaire.

PD-FOG group exhibited decreased volume and surface area of LC compared with both PD-NFOG and HC groups, and decreased CNR_LC_ compared with HC group (Figure 4). Correlation analysis was performed for the volume, surface area and CNR_LC_ with NFOGQ in the PD-FOG patients (Figure 5). Significant correlations were observed between the surface area and CNR of LC and NFOGQ, while no significant correlation was observed between the volume of LC and NFOGQ of the PD-FOG patients.

### 3.3. ICC values and ROC curve of the CNR

ICC values for the inter-rater agreement (SW and YC) were 0.757, 0.872 for the CNR of SN and LC respectively, indicating good inter-rater agreement.

**Figure 6:**
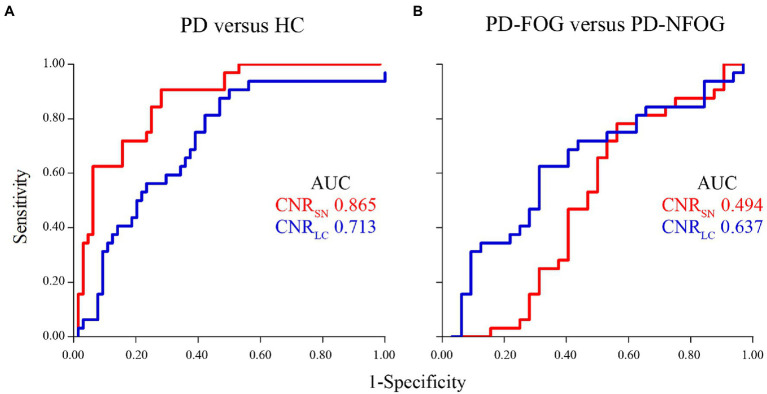
ROC analysis of CNR for differentiating between PD patients and HC **(A)**, between PD-FOG and PD-NFOG **(B)**. AUC, area under the curve; CNR, contrast to noise ratio; ROC, receiver operating characteristics.

ROC analyses indicated that the area under the curve (AUC) in the CNR of SN and LC was 0.865 and 0.713 in PD versus HC, respectively, whereas it was 0.494 and 0.637 in PD-FOG versus PD-NFOG (Figure 6). To discriminate PD patients from the HC, the sensitivity and specificity in the CNR_SN_ was 90.6 and 71.9%, respectively, when the cut-off value for CNR_SN_ was set at 2.101; the sensitivity and specificity in the CNR_LC_ was 90.6 and 50.0%, respectively, when the cut-off value for CNR_LC_ was set at 1.411. No cut-off value was found to discriminate the PD-FOG from the PD-NFOG.

## 4. Discussion

In this study, neuromelanin in SN was significantly decreased in all PD patients, consistent with the findings of previous neuroimaging studies (Reimao et al., 2015a; Schwarz et al., 2017). Obvious symptoms of PD were generally considered to arise, when ≥40% dopaminergic neurons degeneration occurred (Sulzer and Surmeier, 2013a). Even in the early stage of PD, the deficiency of a large number of neuromelanin-containing neurons in SN could be detected. Recent studies had found that the decreased volume and CNR of SN in PD patients were positively correlated with nigrostriatal dopaminergic function detected by SPECT (Ogisu et al., 2013; Kuya et al., 2016; Wang et al., 2018), which was a significant functional neuroimaging method for the diagnosis of PD (Postuma et al., 2015).

The volume and signal intensity of SN were also significantly different between idiopathic PD, parkinsonian syndrome and essential tremor (Reimao et al., 2015b). These results indicated that SN changes observed by NM-MRI could be a biomarker for PD diagnosis. Our results showed that UPDRS III score was negatively correlated with the volume, surface area and CNR of SN. Moreover, illness duration was negatively correlated with the volume and surface area of SN in all PD patients, in agreement with the growing clinical evidence which suggested that NM-MRI changes were associated with disease severity (Fabbri et al., 2017; Schwarz et al., 2017). While, no significant difference in the nigral neuromelanin alterations between the PD-FOG and the PD-NFOG indicated that the loss of neuromelanin might occur across multiple motor features such as tremor and rigidity, but not correlate with FOG symptom.

In addition to the neuromelanin alterations in SN, significant differences of signal intensity in LC were found between PD patients and HC, which was consistent with previous reports (Matsuura et al., 2013; Wang et al., 2018). Although several studies had indicated that degeneration of neuromelanin in LC might display a better diagnostic ability than that in SN (Matsuura et al., 2013), other studies provided conflicting results that reported a lower specificity and sensitivity of LC degeneration (Schwarz et al., 2017). Our results showed the same sensitivity of CNR in SN and LC to discriminate PD patients from HC, but specificity and AUC of SN were higher than those of LC. Previous studies had reported apparent stage-dependent reduction of the neuromelanin-positive areas which represent the volume of SN in PD patients (Kashihara et al., 2011; Ohtsuka et al., 2013), in connection with the known pathological changes with illness duration and progression of disease severity (Sulzer and Surmeier, 2013b). Signal attenuation of SN on NM-MRI was considered to be characteristic neuroimaging findings, reflecting the pathological change of PD directly, and might be more appropriate than LC.

We also showed that the volume and surface area of LC in PD-FOG significantly decreased compared with both PD-NFOG and HC groups. Nuclide molecular functional imaging studies had manifested that PD patients exhibited reduced neuromelanin-containing noradrenergic neurons in LC (Ono et al., 2016). The PD-FOG group showed significantly volume, surface area reductions rather than CNR of LC in further analysis, that might be attributed to monoaminergic neurons *in vivo* was not reflected directly by NM-MRI and differed across FOG subtypes. According to the therapeutic response of levodopa, FOG could be distinguished into subtypes of levodopa reactive and resistant (Giladi et al., 2001; Schaafsma et al., 2003). Previous studies with functional or structural neuroimaging had supported that there were complicated neural mechanisms in various subtypes of FOG, which were associated with lesions in multiple brain regions, in addition to the single lesion of LC (Bartels and Leenders, 2008; Schwarz et al., 2011; Rubino et al., 2014; Vercruysse et al., 2015).

There were several limitations of our study. Firstly, taking into account of ethical and economic considerations, NM-MRI wasn’t compared with traditional imaging technologies of dopamine transporter SPECT or ^123^I-metaiodobenzylguanidine scintigraphy, which were supposed to reliable methods for diagnosis of PD (Jennings et al., 2004; Sawada et al., 2009). Secondly, NM-MRI were operated with relative long time-consuming. Thus, movement artifacts during the process of image acquisition might influence the results. Finally, this was a cross-sectional study with moderate sample size and without pathological confirmation. It was unclear if NM-MRI could distinguish early PD from other parkinsonian syndromes and predict subtypes of FOG. Although the above problems are beyond the scope of our study, further larger sample size prospective studies with different subtypes of FOG are needed.

In conclusion, our study proved that both PD-NFOG and PD-FOG groups, compared with HC, exhibited a decrease of neuromelanin in SN and LC according to NM-MRI. And the measurements of neuromelanin in SN were correlated with the illness duration and severity of motor symptoms. Compared with PD-NFOG, the measurements of neuromelanin in LC of PD-FOG were significantly different and associated with the severity of FOG symptom. The NM-MRI sequence may become a diagnostic biomarker to discriminate neuromelanin changes of SN and LC in PD patients from HC. Although no cut-off value was found to discriminate the PD-FOG from the PD-NFOG according to ROC curve analysis of the CNR_LC_, the cut-off value of CNR_SN_ could differentiate PD patients from HC. Neuromelanin changes of SN presented in both PD-NFOG and PD-FOG groups. While, decreased neuromelanin of LC seemingly appeared more obvious in PD-FOG group.

## Data availability statement

The original contributions presented in the study are included in the article/supplementary material, further inquiries can be directed to the corresponding authors.

## Ethics statement

The studies involving human participants were reviewed and approved by the Ethics Committees of the First Affiliated Hospital of Anhui Medical University (Reference no., Quick-PJ 2021-10-23). The patients/participants provided their written informed consent to participate in this study.

## Author contributions

All co-authors meet the criteria for authorship. SW: conception, organization and execution of research project, analysis of data, and manuscript writing. TW: neurological assessments, study coordinator, and manuscript writing. YC: analysis of data and study coordinator. YY: organization of research project and manuscript review. XC: neurological assessments, subject recruitment, and manuscript review. LW: concept of research project, manuscript review, and final approval of the version to be submitted. All authors contributed to the article and approved the submitted version.

## Funding

This work was supported by grants from the National Natural Science Foundation of China (Grant/Award Numbers: 81971072).

## Conflict of interest

The authors declare that the research was conducted in the absence of any commercial or financial relationships that could be construed as a potential conflict of interest.

## Publisher’s note

All claims expressed in this article are solely those of the authors and do not necessarily represent those of their affiliated organizations, or those of the publisher, the editors and the reviewers. Any product that may be evaluated in this article, or claim that may be made by its manufacturer, is not guaranteed or endorsed by the publisher.

## Footnotes

^1^ http://www.slicer.org
